# Development of a novel prognostic score for 1-year survival in immunotherapy-treated patients with unresectable HCC: the ALIVE-IO score

**DOI:** 10.3389/fimmu.2025.1648248

**Published:** 2025-08-29

**Authors:** Spyridon Pantzios, Orestis Sidiropoulos, Antonia Syriha, Nikolaos Ptohis, Ioannis Skouras, Evgenia Mainta, Dimitris P. Korkolis, Nikolaos Machairas, Georgios C. Sotiropoulos, Ioannis Elefsiniotis

**Affiliations:** ^1^ Academic Department of Internal Medicine-Hepatogastroenterology Unit, General Oncology Hospital of Kifisia “Agioi Anargyroi”, National and Kapodistrian University of Athens, Athens, Greece; ^2^ Department of Interventional Radiology, “G. Gennimatas” General Hospital of Athens, Athens, Greece; ^3^ Department of Radiology, General Oncology Hospital of Kifisia “Agioi Anargyroi”, Athens, Greece; ^4^ Department of Surgery, “Saint Savvas” Hellenic Anticancer Hospital of Athens, Athens, Greece; ^5^ Department of Liver Transplantation and Hepatobiliary Surgery, National and Kapodistrian University of Athens, Athens, Greece

**Keywords:** HCC, immunotherapy, prognostic score, ALIVE-IO score, ALBI, up-to-7 criteria, lymphocyte infiltration

## Abstract

**Background:**

There is a lack in reliable and widely used prognostic scores to predict survival in patients with hepatocellular carcinoma (HCC) receiving immunotherapy. The aim of our study was to develop a prognostic score that could predict 1-year OS in patients with unresectable HCC receiving immunotherapy.

**Methods:**

We studied 100 patients who received 1^st^ line immunotherapy. We did a univariate cox regression analysis to assess which of the patients’ baseline characteristics was associated with OS. Factors strongly associated with OS were used in the multivariate model and their B coefficients were used to produce a normalized score (ALIVE-IO score) that could predict 1-year OS. Internal validation was done using ROC analysis and 10-fold cross-validation. Then, we separated our patients in three risk groups (low, intermediate, high) based on the new score and studied them for their baseline characteristics, response to immunotherapy and OS.

**Results:**

In univariate analysis, significant correlation with OS was found for ALBI grade (p<0.001, HR=2.725), BCLC stage (p=0.031, HR=1.809), macrovascular invasion (p<0.001, HR=2.587), up-to-7 criteria (p<0.001, HR=0.218) and lymphocyte infiltration (p=0.005, HR=0.485). In the multivariate analysis, three factors were significantly correlated with OS; ALBI grade (grade II vs. I, p=0.025, HR=1.946), up-to-7 criteria (beyond vs. within, p=0.001, HR=3.506) and lymphocyte infiltration (no vs. yes, p=0.016, HR=1.889). The ALIVE-IO score was calculated with the contribution of 1 point for ALBI grade II, 2 points for exceeding up-to-7 criteria and 1 point for absence of lymphocyte infiltration. The score had an AUROC of 0.755 for 1-year OS, with 75% sensitivity and 65.4% specificity. We established three risk groups; low (ALIVE-IO: 0-1), intermediate (ALIVE-IO: 2-3) and high (ALIVE-IO: 4). Objective response was reported in 34.8% of patients in the low risk group, compared to 18.5% in intermediate and 4.3% in high risk patients (p=0.031). The median OS of the three groups was 41, 12 and 3 months, respectively (p<0.001). The 1-year OS was 80%, 41% and 16, respectively.

**Conclusion:**

The ALIVE-IO score is a promising tool for predicting 1-year OS in HCC patients undergoing immunotherapy using common laboratory, imaging and histological data frequently used in everyday clinical practice.

## Graphical Abstract

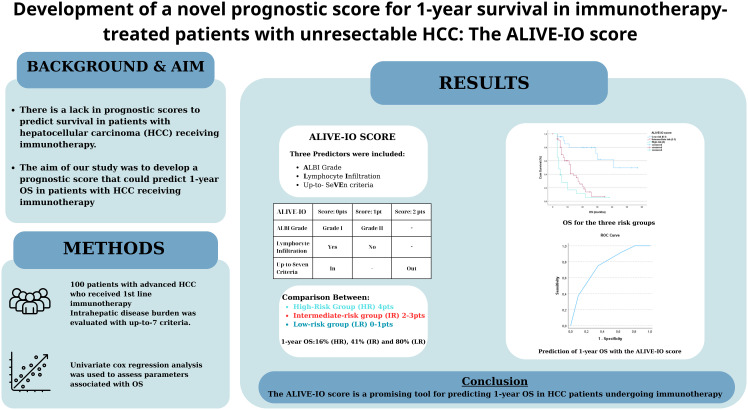

## Introduction

Liver cancer constitutes a difficult clinical entity, as a result of its high mortality and elevated frequency through the last years. It is thought to be the 6^th^ most common type of cancer and the 3^rd^ leading cause of cancer-related mortality worldwide, according to recent data (1). Hepatocellular carcinoma (HCC) is the most common form of liver cancer, accounting for approximately 90% of cases worldwide, the other being cholangiocarcinoma and some rare liver cancer cases (2). Notably, the most common risk factor for HCC development is cirrhosis of the liver, given the fact that approximately 70-75% of all HCC cases arise in cirrhotic patients (3). Chronic hepatitis B (HBV) and chronic hepatitis C (HCV) are the most common etiology of HCC worldwide, although steatotic liver disease (SLD), either resulting from alcohol consumption (alcohol-related liver disease, ALD) or metabolic syndrome (metabolic dysfunction-associated steatotic liver disease, MASLD) has become the most frequent cause of HCC in the USA and the most of the Western countries (4–6).

Unfortunately, the majority of HCC cases are diagnosed in the advanced stage, where potentially curative interventions such as liver resection (LR) or radiofrequency ablation (RFA) are not feasible, resulting in limited treatment options (7). For many years, sorafenib (a multi-targeting Tyrosine-Kinase Inhibitor, TKI) was the only approved systemic treatment for patients with advanced and unresectable HCC, with low response rates and high toxicity (8). Recently, advances in the field of immunology have given rise to new cancer treatments - even in HCC - which blockade immune checkpoints (such as PD-1, PD-L1 and CTLA-4) and activate the host immune response against malignant cells (9). These drugs, either used alone or in combination with other monoclonal antibodies that target VEGF (such as bevacizumab), seem to significantly prolong overall survival (OS) and progression-free survival (PFS) in patients with advanced HCC, compared to sorafenib, which was the previous standard of care (10). Interestingly, these new drugs seem to significantly improve patients’ quality of life, mainly due to their better adverse event profile (11, 12). More precisely, in 2019 the IMBRAVE150 study was the first to show survival benefit in patients with advanced HCC receiving first line atezolizumab-bevacizumab, when compared to the sorafenib-treated patients (11). In 2022 the HIMALAYA study, compared the combination of tremelimumab plus durvalumab (STRIDE) with sorafenib in 1^st^ line HCC systemic treatment and showed superior results in terms of efficacy and safety (12).

These immunotherapy-based regimens have become the new standard of care in the systemic treatment of advanced HCC during the last years, while also being tested in the adjuvant and neoadjuvant setting (13). However, defining which patients will be mostly benefited from these new revolutionary treatments that offer prolonged survival in approximately 25-30% of treated patients, remains one of the most important unmet needs in the field of liver oncology due to the shortage of reliable predictive biomarkers in this setting. Predictive biomarkers that have been tested for patients with HCC receiving immunotherapy regimens, comprise a wide spectrum of tests; blood-derived biomarkers and tissue-derived biomarkers (14). The most commonly studied blood-based biomarkers include alpha-fetoprotein (aFP), C-reactive protein (CRP), Albumin-Bilirubin (ALBI) score, neutrophil-to-lymphocyte ratio (NLR), platelet-to-lymphocyte ratio (PLR), cytokines, circulating-tumor DNA (ctDNA) and circulating tumor cells (CTCs) as recorded in previous studies (14, 15). Tissue-derived biomarkers such as PD-L1 expression, tumor mutational burden (TMB), microsatellite instability (MSI) and tumor infiltrating lymphocytes (TILs) have also been studied (14). The use of these biomarkers in various prognostic scores have been developed through the last years in order to predict survival of immunotherapy-treated HCC patients, but none of these tests have been widely accepted for use in clinical practice and further research is needed in this direction.

The aim of this pilot study was to develop a novel prognostic score for OS in patients with HCC receiving first line systemic therapy with atezolizumab-bevacizumab and tremelimumab-durvalumab, using simple biomarkers and highly available in routine clinical practice, that were independently correlated with OS in these patients.

## Materials and methods

### Patient selection and data

We evaluated a total of one-hundred white European patients diagnosed with intermediate or advanced stage HCC, according to the BCLC classification, who initiated first line immunotherapy for HCC, either with atezolizumab-bevacizumab or with tremelimumab-durvalumab (Single Tremelimumab Regular Internal Durvalumab, STRIDE) in our center. The study of the data was conducted by a single HCC referral center in Athens, Greece (Academic Department of Internal Medicine-Hepatogastroenterology Unit, General and Oncology Hospital of Kifisia “Agioi Anargyroi”, National and Kapodistrian University of Athens). All patients initiated 1^st^ line immunotherapy from September 2020 to January 2025 and were evaluated for all their baseline parameters on the first day of immunotherapy in our center. We recorded demographic factors such as age and sex, along with clinical parameters such as body mass index (BMI), etiology of HCC, presence of diabetes, varices and ALBI grade before the initiation of immunotherapy. Furthermore, data on tumor burden were assessed such as BCLC classification, the presence of macrovascular invasion (MVI) and extrahepatic disease (EHD), categorization depending on the up-to-7 criteria, tumor biomarkers such as aFP and type of 1^st^ line treatment (atezolizumab-bevacizumab or STRIDE). Lastly, we assessed histological parameters such as infiltration by TILs and the morphomolecular classification of HCCs according to the recent categorization, classifying HCCs in two separate categories: proliferative and non-proliferative HCCs (16). The diagnosis of HCC was suspected in all patients with the combined use of serum aFP and cross-sectional imaging techniques such as computed-tomography (CT) and magnetic-resonance imaging (MRI) and confirmed with histological examination of tumor samples either through ultrasound-guided fine needle biopsy or previous liver resection (LR) in patients that HCC rapidly recurred.

Histological assessment of the presence of TILs was performed qualitatively on formalin-fixed, paraffin-embedded (FFPE) tumor samples using hematoxylin and eosin (H&E) staining, as well as immunohistochemical staining for pan-T lymphocyte markers, primarily CD3 and CD8. Immunostaining was carried out using standardized protocols in certified pathology laboratories. Sections (3–4 µm thick) were deparaffinized, rehydrated, and subjected to antigen retrieval before incubation with monoclonal antibodies against CD3, CD4 and CD8. Detection was performed using a peroxidase-based polymer detection system and diaminobenzidine (DAB) as chromogen. The presence of TILs was evaluated qualitatively by two experienced liver pathologists from independent academic centers in Athens, Greece, who were blinded to clinical outcomes. TILs were defined as intra-tumoral lymphocytic aggregates or scattered lymphocytes within the tumor parenchyma, as observed on H&E and confirmed by CD3/CD4/CD8 positivity.

Etiology of HCC was separated into viral and non-viral. Viral HCC etiology comprised of chronic HBV and HCV infections. Patients with chronic HCV infection had received treatment with direct-acting antivirals (DAAs) and presented with undetectable HCV-RNA before the initiation of immunotherapy. Patients with HBV infection had initiated treatment with long-term nucleoside or nucleotide analogues (entecavir, tenofovir) and also had undetectable HBV-DNA before the first immunotherapy. Non-viral HCC etiology consisted of MASLD and/or ALD or both (MetALD) HCC without evidence of active or prior chronic viral hepatitis.

Patients who had received prior systemic therapies were not included in the study. Patients who had received prior locoregional treatment with transarterial chemoembolization (TACE) or RFA (maximum of two sessions for each), were included in the study, along with patients who had previously received LR for primary HCC, had subsequently progressed to a more advanced/unresectable stage and immunotherapy was initiated thereafter, within the first two years after LR. Treatment regimens were as in the prospective trials IMBRAVE150 and HIMALAYA. Atezolizumab was administered at a dose of 1200 mg intravenously combined with 15 mg/kg of intravenous bevacizumab every 21 days and STRIDE was given as single dose (300mg) of intravenous tremelimumab on the 1^st^ cycle, in combination with 1500 mg of durvalumab on the 1^st^ cycle and the same dose every 28 days thereafter, until unacceptable toxicity or loss of clinical benefit. All patients were on a stable dose for both regimens, but treatment could be postponed in case of diagnosis of a grade 3 treatment-related adverse event, and the treatment was permanently discontinued in patients presenting with more severe adverse events. Patients included in the study had received at least 3 cycles of immunotherapy and had at least one tumor assessment imaging at least two months after the initiation of treatment. Tumor assessment was performed with cross-sectional imaging using the mRECIST criteria for HCC (17), and patients were classified in four categories depending on the outcome: complete response, partial response, stable disease and progressive disease. Objective response rates (ORR) contained both complete response and partial response.

### Statistical analysis

For the statistical analysis of our data, we used the IBM SPSS Statistics software version 29.0.2.0. Numerical values are presented with mean values ± standard deviation (SD), whereas categorical values are represented as number and percentages. The independent samples’ t-test was used for the comparison of two different groups of patients in order to find significant differences between continuous variables, while the chi-square test was used to compare categorical variables between two groups. When more than two groups were compared, we used analysis of variance (1-way ANOVA) for normally distributed numerical variables which followed normal distribution, while the Kruskal-Wallis H test was used for non-normally distributed continuous variables and the chi-square test for categorical variables. Normality for all continuous variables was checked using the Kolmogorov-Smirnov test. To construct the prognostic model, we firstly tried to identify key predictors of OS between patients’ studied variables using cox regression univariate analysis for OS, which models the relationship between predictor variables and the hazard of death. We checked for multicollinearity between variables that had a p-value < 0.1 in the univariate cox regression model for OS, using Variance Inflation Factor (VIF). High VIFs indicate that some variables may be too highly correlated with others, leading to potential instability in the regression model. Then we did a cox regression multivariate analysis for OS using all factors that had a p-value < 0.1 in the univariate model and VIFs < 5, indicating no significant multicollinearity. Each of the identified predictors was weighted using their B coefficients from the cox regression multivariate model. These coefficients represent the contribution of each variable to the overall mortality risk. After normalizing the B coefficients, we assessed how many points each parameter contributes to the normalized score. The prognostic score was then computed by adding the weighted scores for each of the variables used in the model, in order to comprise the final formula.

To facilitate clinical decision-making, we stratified patients into risk groups based on their prognostic score and we established three categories (low risk, intermediate risk and high risk) according to the final score. We then did an internal validation using Receiver Operating Characteristic (ROC) analysis with bootstrapping to 1000 for the prediction of 1-year OS, in order to further validate our score and compute confidence intervals (CI) for the ROC curve. Furthermore, a 10-fold cross-validation was done to further assess the generalizability of the score and compute the accuracy of the model and the kappa statistic, in order to predict agreement between predicted and observed outcomes. Finally, we separated our patients in the three risk groups based on the calculated score for each patient and studied these patients for their baseline characteristics, treatment response and OS, using Kaplan-Meier curves. The log-rank test was used to compare survival between the three groups. In case were one or more variables differed statistically significantly between the three groups, in order to assess the possible impact of the prognostic score to OS, we used a cox regression multivariate analysis for OS after adjusting for all potential confounders. P-values lower than 0.05 were considered statistically significant. Cox-regression analysis results are presented using p-values, hazard ratios (HR) and 95% CI for HR.

### Informed consent

Patients who were included in the current study gave written informed consent before the performing of the biopsy and before immunotherapy administration. A separate written informed consent was signed by all participants regarding the use of their data anonymously for scientific purposes. The study protocol was in accordance with the Declaration of Helsinki for human trials and was approved by the Ethics Committee and the Scientific Board of our hospital.

## Results

In total, one-hundred patients with HCC receiving first-line immunotherapy were studied in our cohort. Baseline characteristics of the study population are presented in **Table 1**. Most patients were males (82/100, 82%), with a mean age of 68 years (SD 10.2) and a mean BMI of 26.87 (SD 4.7). Viral HCC etiology was documented in 41 patients (41%), while the rest were considered non-viral (59%). Diabetes and varices were present in 32 (32%) and 27 (27%) patients, respectively. Most of patients were categorized as ALBI grade II (57/100, 57%), while 43 patients had ALBI grade I (43%). At immunotherapy initiation, 60 patients were categorized in the BCLC-C stage (60%) and 40 patients in the BCLC-B stage (40%). BCLC-C patients had MVI in 51.7% of cases (31/60) and EHD in 50% of cases (30/60). Thirty patients (30%) were within up-to-7 criteria considering their intrahepatic disease, irrespective of the presence of MVI and/or EHD, and fifty-three patients (53%) were found to have tumor infiltration by TILs histologically. Fifty-three patients were categorized according to the morphomolecular histological classification as non-proliferative HCCs (53%), while the other 47% was considered to have proliferative HCC. A total of 54 patients had received prior non-systemic treatment (21 patients had liver resection, 24 had RFA and 38 had previous TACE). Seventy-five patients received atezolizumab-bevacizumab (75%), and 25 patients received STRIDE (25%). The median OS in all patients from the start of immunotherapy was 7.5 months at the time of data extraction and the median PFS was 5 months.

**Table 1:** Baseline characteristics of the study population.

Baseline characteristics		Total number of patients (N=100)	
		n	(%)
Gender	Males	82	82,0%
	Females	18	18,0%
Age (years, mean, SD)		68,00	10,20
BMI (kg/m2, mean, SD)		26,87	4,70
Etiology	Viral	41	41,0%
	Non-viral	59	59,0%
Diabetes	No	68	68,0%
	Yes	32	32,0%
Varices	No	73	73,0%
	Yes	27	27,0%
ALBI grade	Grade I	43	43,0%
	Grade II	57	57,0%
BCLC stage	Stage B	40	40,0%
	Stage C	60	60,0%
MVI	No	69	69,0%
	Yes	31	31,0%
EHD	No	70	70,0%
	Yes	30	30,0%
Up-to-7 criteria	Beyond	70	70,0%
	Within	30	30,0%
aFP (ng/mL, median, IQR)		57,2	1647,95
Lymphocyte infiltration	No	47	47,0%
	Yes	53	53,0%
Morphomolecular classification	Non-proliferative	53	53,0%
	Proliferative	47	47,0%

BMI, body mass index; SD, standard deviation; ALBI grade, Albumin-Bilirubin grade; BCLC, Barcelona Clinic Liver Cancer; MVI, macrovascular invasion; EHD, extrahepatic disease; aFP, alpha-fetoprotein; IQR, interquartile range.

We then assessed all parameters for their separate association with OS, using univariate cox-regression analysis for OS and only factors that had p-values < 0.1 were used in the multivariate analysis (**Table 2**). Factors that emerged to have a significant correlation with OS were ALBI grade (p<0.001, HR=2.725, 95% CI: 1.585 – 4.685), BCLC stage (p-value=0.031, HR=1.809, 95% CI: 1.057 – 3.098), MVI (p<0.001, HR=2.587, 95% CI: 1.537 – 4.352), up-to-7 criteria (p<0.001, HR=0.218, 95% CI: 0.105 – 0.455) and tumor lymphocyte infiltration (p=0.005, HR=0.485, 95%CI: 0.293 – 0.805). Baseline aFP values were not statistically significantly correlated with OS in the univariate model (p=0.088) but were included in the multivariate analysis as a widely accepted biomarker. Before proceeding with the multivariate cox regression model, we firstly checked for multicollinearity issues in the aforementioned variables using VIF values < 5 and did not find any significant multicollinearity problems. Therefore, we proceeded to the multivariate cox-regression model after recoding the variables of “up-to-7 criteria” and “lymphocyte infiltration”, so that being beyond the up-to-7 criteria and having no lymphocyte infiltration would be considered high risk according to the results of the univariate cox-regression model (**Table 3**). In the multivariate cox-regression we found that only three factors were significantly correlated with OS; ALBI grade (grade II vs. grade I, p=0.025, HR=1.946, 95% CI: 1.087 – 3.485), up-to-7 criteria (beyond vs. within, p=0.001, HR=3.506, 95% CI: 1.630 – 7.538) and tumor lymphocyte infiltration (no vs. yes, p=0.016, HR=1.889, 95% CI: 1.123 – 3.175).

**Table 2:** Univariate cox-regression analysis for OS.

Potential risk factors	P-value	HR	95,0% CI for HR	
			Lower	Upper
Age	0,977	1,000	0,977	1,024
BMI	0,725	0,991	0,940	1,044
Etiology	0,322	3,120	1,765	5,514
Diabetes	0,857	0,952	0,558	1,624
Varices	0,135	1,517	0,879	2,619
ALBI grade	<0,001	2,725	1,585	4,685
BCLC stage	0,031	1,809	1,057	3,098
Macrovascular invasion	<0,001	2,587	1,537	4,352
Extrahepatic disease	0,416	1,245	0,734	2,110
Up-to-7 criteria	<0,001	0,218	0,105	0,455
aFP	0,088	1,000	1,000	1,000
Lymphocyte infiltration	0,005	0,485	0,293	0,805
Morphomolecular classification	0,307	1,298	0,787	2,142
Immunotherapy regimen	0,853	1,065	0,549	2,066

CI, confidence interval; HR, hazard ratio; BMI, body mass index; ALBI grade, Albumin-Bilirubin grade; BCLC, Barcelona Clinic Liver Cancer; aFP, alpha-fetoprotein.

**Table 3:** Multivariate cox-regression analysis for OS.

Risk factors	B-coefficient	p-value	HR	95,0% CI for HR	
				Lower	Upper
ALBI grade (grade II vs. grade I)	0,685	0,018	1,984	1,127	3,492
BCLC stage	0,457	0,110	1,579	,902	2,763
aFP	0,000	0,556	1,000	1,000	1,000
Up-to-7 criteria (beyond vs. within)	1,293	0,001	3,644	1,735	7,657
Lymphocyte infiltration (no vs. yes)	0,632	0,017	1,881	1,118	3,164

CI, confidence interval; HR, hazard ratio; ALBI grade, Albumin-Bilirubin grade; aFP, alpha-fetoprotein.

To compose the prognostic score after identifying the three predictors of OS, we used the B coefficients of each parameter, derived from the multivariate cox regression model. To create a more practical scoring system, we normalized the B coefficients by dividing each value by the ALBI B coefficient (**Table 4**). As a result, the normalized score was calculated with the contribution of 1 point for ALBI grade II (0 points for patients with ALBI grade I), 2 points for exceeding up-to-7 criteria (0 points for patients within the up-to-7 criteria) and finally 1 point for patients with absence of lymphocyte infiltration (0 points for presence of intratumoral lymphocytes). The new constructed score was named after the variables from which it consisted of as follows; ALBI grade, Lymphocyte Infiltration, up-to-seVEn criteria (ALIVE-IO score). The ALIVE-IO prognostic score was then computed according to the weighted scores for each of the three variables: ALIVE-IO score = (1 × ALBI) + (2 × up-to-7 criteria) + (1 × lymphocyte infiltration), ranging from 0 to 4. In total, 10 patients had a score of 0 (10%), 13 had a score of 1 (13%), 23 patients had a score of 2 (23%), 31 patients had a score of 3 (31%) and 23 patients had a score of 4 (23%). Using the total score, we established three categories; the low-risk group (score: 0-1), the intermediate-risk group (score: 2-3) and the high-risk group (score: 4). As noted before, 23% of patients were categorized in the low-risk group, 54% in the intermediate-risk group and 23% in the high-risk group.

**Table 4:** B coefficients of the three parameters significantly associated with OS and normalization to points for the ALIVE-IO score.

Variable	B coefficient	B ÷ 0.685	Normalized score
ALBI grade II	0.685	1.00	1 point
Beyond up-to-7 criteria	1.293	1.89	2 points
Absence of lymphocyte infiltration	0.632	0.92	1 point

ALBI grade, Albumin-Bilirubin grade.

**Figure 1:**
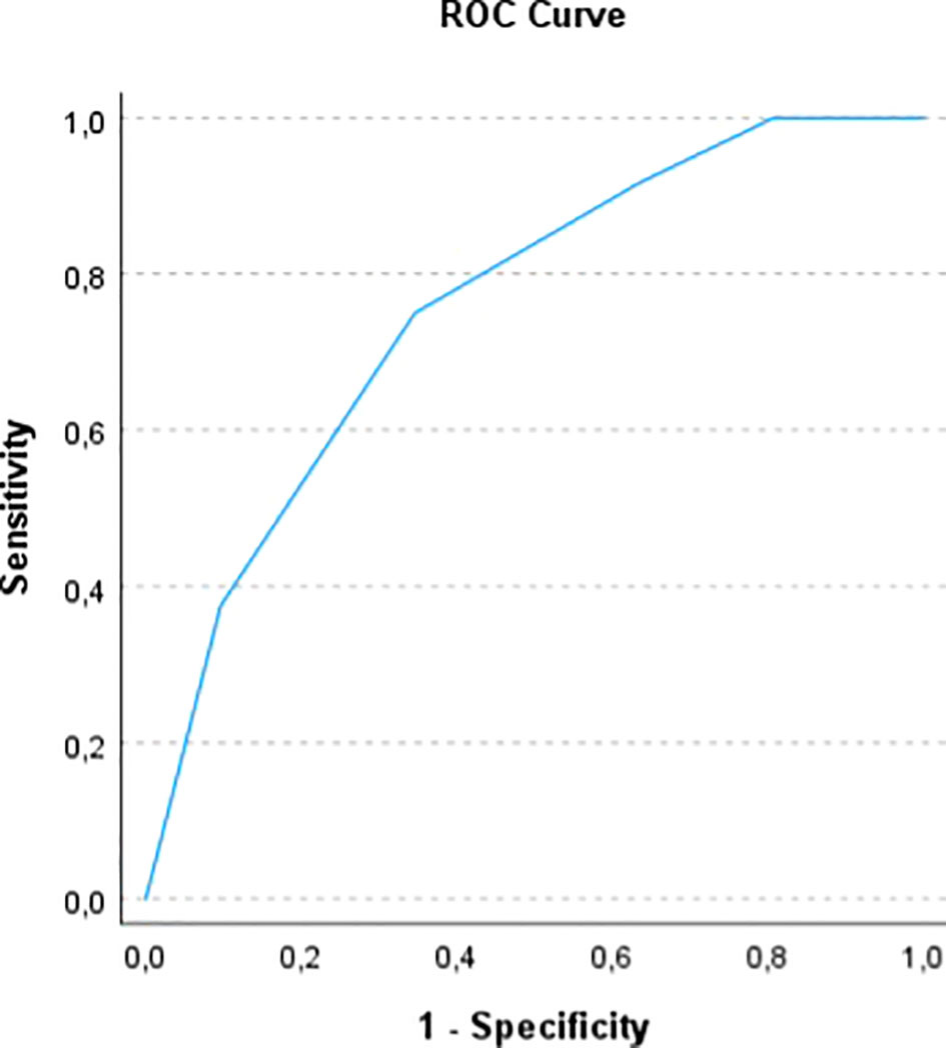
ROC curve for the prediction of 1-year OS with the ALIVE-IO score.

In order to assess the prognostic power of the computed ALIVE-IO score, we did an internal validation of the model. Firstly, a time-dependent ROC analysis with bootstrapping to 1000 was done, which showed an area under the ROC curve (AUROC) of 0.755 (95% CI: 0.6618 – 0.8444, **Figure 1**) for 1-year mortality, with 75% sensitivity, 65.4% specificity, 66.7% positive predictive value (PPV) and 73.9% negative predictive value (NPV). We also did a 10-fold cross-validation to further assess the generalizability of the score and the results indicated an accuracy of 69.7% and a Kappa statistic of 0.4, indicating moderate agreement between predicted and observed outcomes.

**Figure 2:**
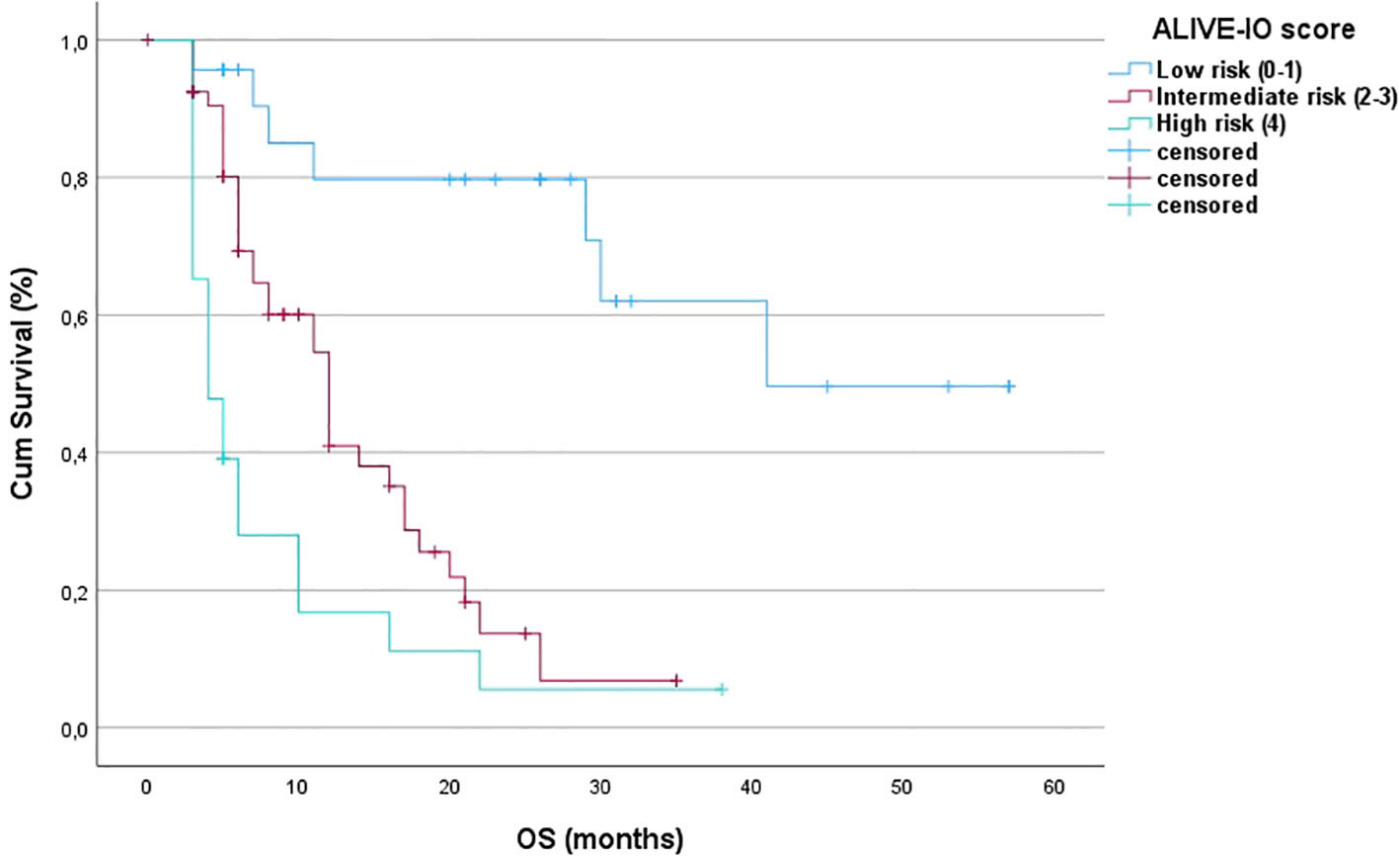
Kaplan-Meier OS curves for the three risk groups (OS, overall survival; Cum survival, cumulative survival).

After the internal validation, we stratified our dataset in groups based on their cumulative risk according to the ALIVE-IO score. Three groups were constructed: the low-risk group (n=23), the intermediate-risk group (n=54) and the high-risk group (n=23). These groups were compared for their baseline characteristics as shown in **Table 5**. As expected, the three groups differed statistically significantly for the values of baseline ALBI, up-to-7 criteria and lymphocyte infiltration. More precisely, we observed that all high-risk patients exhibited ALBI grade II, absence of intratumoral lymphocyte infiltration and exceeded the up-to-7 criteria, while all low-risk patients were within the up-to-7 criteria. Furthermore, the three groups presented statistically significant differences regarding the presence of MVI (p=0.006), with almost all except for two patients in the low-risk group having absence of MVI, and aFP values (p=0.024) with patients at high risk presenting higher aFP values compared to patients in the intermediate and low risk groups. Concerning tumor response according to the mRECIST criteria, we observed a statistically significant difference between the three groups, with low-risk patients having 34% ORR compared to 18.5% in the intermediate-risk group and 4.3% in the high-risk group (p=0.031). We then proceeded to a Kaplan-Meier survival analysis for OS, which showed significant differences in OS between the three groups (p<0.001 by log-rank test, **Figure 2**). The median OS of the three groups was 41, 12 and 3 months for low, intermediate and high-risk patients, respectively. The 1-year OS was 80%, 41% and 16%, the 2-year OS was 80%, 14% and 6% and the 3-year OS was 62%, 7% and 6% for low, intermediate and high-risk groups, respectively. We then conducted a multivariate cox-regression analysis using all parameters for which the three groups were not comparable (including only MVI, aFP and the ALIVE-IO score and excluding its three substances) in order to further assess the effect of categorization by the ALIVE-IO score in OS (**Table 6**). The multivariate analysis showed that the categorization by the ALIVE-IO score was the only factor that was associated statistically significantly with OS, with intermediate-risk patients having a HR of 3.037 (95% CI: 1.039 – 8.876, p=0.042) and high-risk patients having a HR of 7.751 (95% CI: 2.504 – 23.995, p<0.001) for worse OS, both compared to low-risk patients and independently from the presence of MVI and aFP. When only the intermediate and high-risk patients were studied, we found that the intermediate-risk patients had a HR of 0.459 (95% CI: 0.262 – 0.803, p-value=0.006) compared to high-risk patients.

**Table 5:** Baseline characteristics of the three risk groups based on the ALIVE-IO score.

Baseline characteristics		Total number of patients (N=100)						p-value
		High-risk (n=23)		Intermediate-risk (n=54)		Low-risk (n=23)		
		n	%	n	%	Low	%	
Gender	Male	19	82,6%	43	79,6%	20	87,0%	0,743
	Female	4	17,4%	11	20,4%	3	13,0%	
Age (years, mean, SD)		69,65	9,30	67,07	11,22	68,48	8,64	0,583
BMI (kg/m2, mean, SD)		27,19	4,50	26,04	4,48	28,51	5,15	0,100
Etiology	Viral	9	39,1%	22	40,7%	10	43,5%	0,955
	Non-viral	14	60,9%	32	59,3%	13	56,5%	
Diabetes	No	15	65,2%	36	66,7%	17	73,9%	0,781
	Yes	8	34,8%	18	33,3%	6	26,1%	
Varices	No	14	60,9%	41	75,9%	18	78,3%	0,321
	Yes	9	39,1%	13	24,1%	5	21,7%	
ALBI grade	Grade I	0	0,0%	28	51,9%	15	65,2%	<0,001
	Grade II	23	100,0%	26	48,1%	8	34,8%	
BCLC stage	Stage B	5	21,7%	25	46,3%	10	43,5%	0,122
	Stage C	18	78,3%	29	53,7%	13	56,5%	
MVI	No	11	47,8%	37	68,5%	21	91,3%	0,006
	Yes	12	52,2%	17	31,5%	2	8,7%	
EHD	No	15	65,2%	40	74,1%	15	65,2%	0,629
	Yes	8	34,8%	14	25,9%	8	34,8%	
Up-to-7 criteria	Beyond	23	100,0%	47	87,0%	0	0,0%	<0,001
	Within	0	0,0%	7	13,0%	23	100,0%	
aFP (ng/mL, median, IQR)		1177	26500	122,1	1613,7	10,3	235,3	0,024
Lymphocyte infiltration	No	23	100,0%	19	35,2%	5	21,7%	<0,001
	Yes	0	0,0%	35	64,8%	18	78,3%	
Morphomolecular classification	Non-proliferative	12	52,2%	28	51,9%	13	56,5%	0,928
	Proliferative	11	47,8%	26	48,1%	10	43,5%	
Immunotherapy	A/B	18	78,3%	39	72,2%	18	78,3%	0,785
	STRIDE	5	21,7%	15	27,8%	5	21,7%	
mRECIST response	Complete response	0	0,0%	3	5,6%	5	21,7%	0,045
	Partial response	0	0,0%	7	13,0%	3	13,0%	
	Stable disease	4	17,4%	8	14,8%	4	17,4%	
	Progressive disease	19	82,6%	36	66,7%	11	47,8%	
ORR	No	22	95,7%	44	81,5%	15	65,2%	0,031
	Yes	1	4,3%	10	18,5%	8	34,8%	

BMI, body mass index; SD, standard deviation; ALBI grade, Albumin-Bilirubin grade; BCLC, Barcelona Clinic Liver Cancer; MVI, macrovascular invasion; EHD, extrahepatic disease; aFP, alpha-fetoprotein; IQR; interquartile range; A/B, atezolizumab-bevacizumab; STRIDE, tremelimumab-durvalumab; ORR, objective response rates.

**Table 6:** Cox-regression analysis for OS between the three risk groups after adjusting for MVI.

Variables	p-value	HR	95,0% CI for HR	
			Lower	Upper
Low risk (reference)	<0,001			
Intermediate vs. low risk	0,042	3,037	1,039	8,876
High vs. low risk	<0,001	7,751	2,504	23,995
MVI	0,079	1,714	0,940	3,125
AFP	0,434	1,000	1,000	1,000

CI, confidence interval; HR, hazard ratio; MVI, macrovascular disease; AFP, alpha-fetoprotein.

## Discussion

Clinicians treating unresectable HCC patients with immunotherapy are in lack of widely accepted and used prognostic scores for the prediction of clinical outcomes and survival surrogates in this setting. As mentioned before, some prognostic scores have already been demonstrated, most of which assess various biomarkers with or without their combination with HCC burden and/or liver disease severity that frequently impacts survival in patients with HCC. As a result, we believe that it is very crucial to summarize some of the evidence we currently have on HCC biomarkers and prognostic scores, comparing them with the new score that was developed and proposed from our study and coming to our conclusions.

### Blood-derived biomarkers in HCC immunotherapy

The most frequently used blood-derived biomarker that is highly available and widely used, is serum aFP, which has been shown to be correlated with HCC aggressiveness, microvascular invasion and high histological grade (18–20). Although data from a wide meta-analysis of retrospective studies reported that higher levels of baseline aFP are associated with increased hazard of death and disease progression in immune-checkpoint inhibitor (ICI)-treated patients (15), the subgroup analysis of the prospective ICI studies for HCC showed controversial results, with IMBRAVE150 study showing survival benefit for patients with lower baseline aFP levels. On the contrary, studies such as HIMALAYA, CheckMate-459, LEAP-002 and more recently the CheckMate-9DW trial, showed survival benefit for patients with elevated aFP (12, 21–23). Furthermore, not only baseline aFP levels, but also aFP changes during immunotherapy have been shown to be correlated with survival and response, suggesting its possible use as a dynamic biomarker during treatment (24–26). However, a common problem with aFP in patients with HCC is that up to approximately 40% of patients may not express aFP before initiation of immunotherapy and as a result reductions in aFP levels cannot be used for the identification of possible early responders (27).

Other blood-based HCC biomarkers that have been extensively studied include NLR and PLR, calculated using complete blood count with differential results. Data from several studies show that not only baseline, but also sequential measurements of NLR and PLR might be associated with poorer survival outcomes and treatment response rates, a finding that was also reported previously from our group (15, 28–30). Although they are two easily obtained biomarkers, common problems are the absence of specific cut-off values that can be widely used, possible changes in these values during active infections and the great heterogeneity of their values between cirrhotic and non-cirrhotic patients. Furthermore, other blood-derived biomarkers such as cytokines (IL-6, IL-8 and TGF-β among others), ctDNA and CTCs have also been studied in some patients with HCC receiving immunotherapy, but their limited availability and high cost, could be a major drawback – at least for the time being – for using them in predictive scores for immunotherapy in advanced HCC (31–35).

### Tumor-derived biomarkers in HCC immunotherapy

Apart from blood-based biomarkers, there is also a wide variety of tumor-derived biomarkers that have been evaluated for prediction of immunotherapy response. Firstly, high histological expression of PD-L1 has been shown to correlate with better disease response in various studies, including prospective trials of immunotherapy in HCC, while others failed to prove such correlations (11, 36–38). A major disadvantage of PD-L1 measurement through immunohistochemistry is the wide discrepancies that can be noted by different pathologists assessing clinical samples with potentially different assays and varying cut-offs reported (39). Other tumor-based biomarkers such as TMB and MSI have also been studied, though with low samples and inconsistent results across studies (40–43). Although these biomarkers have already been extensively used in other solid tumors, HCC appears to have low TMB and MSI, therefore making their evaluation unsafe in this subgroup of patients (44, 45).

Another tumor feature that has been widely studied is the presence of TILs, mainly T-helper and cytotoxic T-lymphocytes (CTLs). The rationale for their study in the context of HCC, is that their intratumoral or peritumoral presence can greatly affect the outcome of immunotherapy, as they can interact with tumor-derived antigens and initiate or maintain an anti-tumoral immunological response (46). It has been proposed that HCC patients with tumors which have high infiltration by TILs have higher survival rates and better response to immunotherapy (40). The understanding of these mechanisms of tumor-immune system interaction, has led further research to propose a new classification for HCC immunological profiling, featuring inflamed and non-inflamed classes (47). Furthermore, emerging data from RNAseq analysis on atezolizumab-bevacizumab treated HCC patients regarding the previous classification, revealed that patients with inflamed HCC have better response rates than patients with non-inflamed HCC, suggesting the presence of TILs as a potential biomarker (48).

### The effect of liver disease severity and tumor burden in HCC immunotherapy

As noted before, patients with HCC represent a unique clinical subset, as a result of the co-existence of two distinct factors that can both affect OS, namely liver cirrhosis and HCC itself (3). Liver disease severity can be assessed through various parameters, such as albumin, bilirubin and prothrombin time and multiple scores have been used for that purpose, including the Child-Pugh score, Model for End-stage Liver Disease (MELD) with its variations and Albumin-Bilirubin (ALBI) score (49, 50). In particular, ALBI grade is more efficient in defining liver disease severity in patients with HCC compared to MELD and Child-Pugh scores, given its applicability in both cirrhotic and non-cirrhotic patients. Moreover, ALBI pre-treatment values have been correlated previously with OS in patients with HCC receiving immunotherapy, as patients with lower ALBI grades experience better survival outcomes (51).

Moreover, tumor burden plays a significant role in the survival of patients with HCC and thus it is included in all staging algorithms such as the Barcelona Clinic Liver Cancer (BCLC) classification (52). According to the updated 2022 BCLC classification, patients receiving systemic therapy are not limited to BCLC-C stage, but can also be classified in earlier stages, mainly BCLC-B stage with diffuse/infiltrative/massive disease (BCLC-B3) not amenable to locoregional treatments. As a result, the intrahepatic tumor burden could play an important role in determining the survival of patients under systemic therapy, as reported in some previous studies (53, 54). The up-to-7 criteria have been generally used in the selection of HCC patients for liver transplantation and is a valuable tool in assessing the intrahepatic tumor burden (55). Recently, they have been assessed in combination with the ALBI grade for predicting outcomes in patients receiving lenvatinib or immunotherapy for advanced HCC (56).

### Prognostic scores proposed for HCC immunotherapy

Through the last years, several prognostic scores have been proposed in order to predict outcomes in patients with HCC receiving immunotherapy, as this is a major unmet need in the treatment of HCC. Firstly, in 2022 the CRAFITY score was introduced, which included baseline values of CRP and aFP for patients with HCC receiving immunotherapy (57). The study evaluated 292 patients who were separated in three risk groups according to the points attributed to each patient (low, intermediate, high risk). Patients with low CRAFITY score had better OS and better responses to immunotherapy than patients in the intermediate risk group, with high risk patients having the worst outcomes. The model worked well for both the training and the validation cohort. Although the CRAFITY score consists of two non-expensive and easily obtained blood tests such as CRP and aFP, it is critical to mention that the study was retrospective and the patients who were studied could have received immunotherapy for HCC not only in the 1^st^ line (approximately 40% of cases), but also in later lines, suggesting high heterogeneity of the study group. Furthermore, the authors clearly suggest caution with the evaluation of CRP as it is generally associated with cardiovascular diseases, which can also lead to worse OS, along with the HCC presence (58). We would also suggest cautious evaluation of CRP in HCC patients, the majority of whom are cirrhotic and could possibly have relatively lower baseline CRP values attributed to the severity of the underlying liver disease, leading to false negative results (59).

Another proposed risk score for HCC patients receiving immunotherapy was the hepatocellular carcinoma immune prognosis score (HCIPS), which was developed using albumin and thrombin time from a retrospective single-center study from China, in 151 patients who received atezolizumab-bevacizumab or camrelizumab-apatinib at the context of a clinical trial (60). The score had an AUROC of 0.609 (95% CI: 0.519-0.699), suggesting low to intermediate predictive accuracy. It is critical to mention that although the score distinguished patients between two groups according to a cut-off value of 0.64, with patients with HCIPS < 0.64 having the worst PFS and OS, this study did not evaluate patients for response to immunotherapy and – as in our study – no external validation was done. Furthermore, albumin and thrombin time are closely associated with liver disease severity and in the multivariate analysis of the study for OS, liver cirrhosis was almost statistically significant (p=0.06) suggesting possible significant values with greater samples. Furthermore, BCLC stage also emerged as a significant factor, which along with HCIPS had a significant correlation with OS.

In March 2024, a multi-center study from Italy and Japan developed the α-FAtE score as a predictive score for immunotherapy-treated HCC patients (61). This study retrospectively evaluated 543 patients treated with atezolizumab-bevacizumab (204 patients) or lenvatinib (339 patients) and consisted of baseline aFP, alkaline phosphatase (ALP) and eosinophil count. Patients in the high risk group (low score) had worse OS and lower time-to-progression (TTP) compared to patients in the low risk group (high score). The score had an AUROC of 0.794, suggesting moderate-to-good predictive accuracy. However, not all patients in the study had baseline ALP measurements, decreasing the number of atezolizumab-bevacizumab studied patients in 153 for the development of the model and possibly reducing its reproducibility.

Furthermore, in November 2024 another risk score was proposed for patients with HCC treated with combined hepatic arterial infusion chemotherapy (HAIC) and anti-PD-L1 immunotherapy, the AFCRPLITY score, which used aFP, CRP and PLR for the stratification of patients into risk groups (62). Although the model stratified patients in four groups and showed that the low risk groups had better disease control rate (DCR) and PFS compared to the high risk groups, while also having an external validation dataset, it may have certain limitations related to CRP and PLR, as mentioned above.

More recently, the CABLE score was developed, with the retrospective study of 683 patients from Europe and the USA with HCC, who received 1^st^ line atezolizumab and bevacizumab. The score consisted of CRP, albumin, bilirubin, lymphocytes, performance status and extrahepatic disease (63). The score had a very good predictive accuracy with an AUROC of 0.79 and was superior to other prognostic scores, such as ALBI for predicting OS especially in the first 9 months of immunotherapy. Furthermore, patients were stratified in three risk groups with statistically significant differences regarding their OS and the model worked well in both cohorts (training and validation sets). Some more risk scores have also been developed, such as the HCC-GRIm score and the ABE index, which included some of the most common studied laboratory biomarkers, with equal diagnostic accuracy and same disadvantages as the previous models (64, 65).

It is crucial to mention that all the aforementioned risk prediction scores use common laboratory parameters, without exploring their association with the tumor immune microenvironment and the possible prognostic role of intratumoral lymphocyte infiltration. To our knowledge, this is the first prognostic score for immunotherapy treated HCC patients that combines common laboratory tests with histological data and intrahepatic disease burden. According to our results, the newly proposed ALIVE-IO score demonstrated robust predictive accuracy through internal validation with an AUROC of 0.755 indicating moderate-to-good predictive performance for 1-year OS in immunotherapy treated HCC patients. It also seems that the intrahepatic burden of disease – which in our study was assessed using the up-to-7 criteria – could possibly have an impact in the OS of these patients and it should be clearly identified as a potential prognostic factor that, in combination with other biomarkers, could predict survival or even disease response in some patients. Furthermore, it is critical to underline that all patients received immunotherapy in the 1^st^ line setting and not as a subsequent line of systemic therapy, and treatment contained not only atezolizumab and bevacizumab – for which all the aforementioned scores were studied – but also the STRIDE combination, which is a newer immunotherapy regimen in HCC and clearly under-represented in all the currently proposed HCC risk scores.

The ALIVE-IO prognostic score serves as a clinically applicable tool for predicting 1-year survival in unresectable HCC patients undergoing immunotherapy. Its utility lies in its simplicity and ability to provide quick, reliable risk stratification based on easily obtainable laboratory, imaging and histological data. Its key advantages are its objective risk assessment, as it is derived from an objective cox regression model, ensuring that the scores reflect the actual risk rather than subjective clinical judgement. The score can aid clinicians in making critical treatment decisions, such as whether to continue – or even initiate – immunotherapy or consider other therapeutic options based on the patient’s predicted survival. The categorization of patients into low, intermediate and high risk groups allows clinicians to tailor treatments more precisely and allocate resources efficiently.

### Limitations of the study

While the ALIVE-IO prognostic score offers several strengths, it is important to acknowledge certain limitations. First, the retrospective nature of our study should be clearly mentioned, along with the moderate sample of patients. Second, the score was validated only internally, using the ROC analysis and 10-fold cross-validation methods and there was no validation set. The absence of external validation with independent datasets limits its generalizability. Further studies in independent cohorts are necessary to confirm the robustness and applicability of the score in different patient populations. Third, the current model is focused exclusively on OS. To enhance the score’s clinical utility, further validation is needed to determine its prognostic value for progression-free survival (PFS) and other clinical outcomes. Fourth, while the model includes important clinical variables, it does not incorporate other potentially relevant biomarkers (e.g., genetic mutations, inflammatory cytokines) that could clearly improve its predictive performance, while also increasing its cost, or common laboratory parameters (such as aFP, NLR, PLR, CRP) which are widely used in most of the other proposed risk scores. Fifth, the ALIVE-IO score simplifies the complex clinical factors into a single number, but it may not account for all nuances of patient conditions. Clinicians should use it as an adjunct to their clinical judgment rather than a sole determinant in decision-making. Sixth, tumor lymphocyte infiltration was assessed only qualitatively and not quantitatively whereas specific lymphocyte subsets were not reported. Furthermore, lymphocyte infiltration of each case was evaluated by different pathologists, indicating a relative heterogeneity in the assessment of the presence or absence of TILs, as observed in everyday clinical practice. Finally, we should keep in mind that the assessment of liver tumor lymphocyte infiltration through imaging-guided biopsy could possibly lead to underestimation of lymphocyte infiltration compared to LR-derived biopsies. Nonetheless, the score is simple and feasible and underlines the significance of widely accepted laboratory parameters that assess liver function, imaging data that define intrahepatic tumor burden and histological data which are demanded in the vast majority of cases for HCC diagnosis, to predict survival in unresectable HCC patients treated with 1^st^ line immunotherapy.

In conclusion, the ALIVE-IO prognostic score is a promising new tool for predicting 1-year OS in unresectable HCC patients undergoing first-line immunotherapy. Based on key variables like ALBI grade, disease burden according the up-to-7 criteria, and intratumoral lymphocyte infiltration, the proposed score demonstrated good predictive performance. Despite the aforementioned limitations, the ALIVE-IO prognostic score holds potential as an integral part of clinical decision-making, helping stratify patients and guide therapeutic strategies more effectively in the era of personalized immunotherapy.

## Data Availability

The raw data supporting the conclusions of this article will be made available by the authors, without undue reservation.
